# Clinical Management of Bile Duct Diseases: Role of Endoscopic Ultrasound in a Personalized Approach

**DOI:** 10.3390/jpm11010001

**Published:** 2020-12-22

**Authors:** Torsten Beyna, Christian Gerges

**Affiliations:** Department of Gastroenterology and Therapeutic Endoscopy, Evangelisches Krankenhaus Düsseldorf, 40217 Düsseldorf, Germany; christian.gerges@evk-duesseldorf.de

**Keywords:** endoscopic ultrasound, EUS, biliary diseases, biliary stones, indeterminate biliary stricture, EUS-guided fine needle biopsy, biliary drainage, failed ERCP

## Abstract

Biliary diseases are common, but clinical symptoms are often unspecific and direct access and visualization of the biliopancreatic system for diagnostic purpose is difficult. In the last decades endoscopic ultrasound (EUS) has become a primary method in the gastrointestinal tract. It significantly changed the role of endoscopy in diagnostic imaging in the gastrointestinal tract and adjacent organs. EUS has become an effective diagnostic tool in biliary stone disease as well as in the diagnosis of indeterminate biliary strictures. Furthermore, an EUS-directed transmural approach emerged as a safe and effective alternative to ERCP in patients requiring biliary drainage, in particular as a backup method if standard ERCP-approach fails. Development of new techniques, specific accessories and stents during the last decade led to an enormous step forward in terms of efficacy and safety of an EUS-directed approach. In the current article technical and clinical aspects of EUS-guided diagnostic and therapeutic approaches in different clinical indications will be discussed together with a review of the available data.

## 1. Introduction

**Figure 1:**
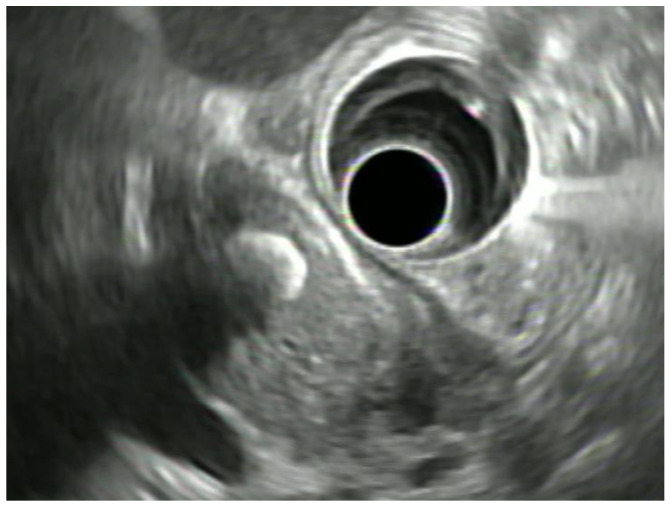
Radial EUS scan of the distal CBD from the second portion of the duodenum showing a stone in front of the papilla. CBD—common bile duct.

Biliary diseases are common, but clinical symptoms are often unspecific and direct access and visualization of the biliopancreatic system for diagnostic purpose is difficult. The diagnostic approach to bile duct diseases often poses a challenge to a multidisciplinary team of gastroenterologists, endoscopists, radiologists and surgeons and there is a massive reliance on multimodal imaging-techniques to obtain the correct diagnosis: Transabdominal ultrasound is commonly used as a primary, non-invasive investigative tool with computed tomography (CT) and magnetic resonance imaging (MRI) and MR-cholangiopancreatography (MRCP), respectively, utilized for more detailed analyses. Endoscopic retrograde cholangiopancreatography (ERCP) may be used for diagnostic purposes, but is increasingly restricted to therapeutic indications due to the risk of post-ERCP pancreatitis (PEP) [1]. In the last decades endoscopic ultrasound (EUS) has become a primary tool for diagnosis and staging (D&S) of neoplasms in the gastrointestinal tract. It significantly changed the role of endoscopy in diagnostic imaging in- and outside the gastrointestinal tract. EUS combines the endoscopic visualization of the gastrointestinal lumen with the ability to ultrasonographically display the layers of the GI wall and the surrounding structures with high resolution. EUS allows for precise real time images of important internal organs alongside the GI tract (i.e., pancreas and the biliary system) which is mandatory for fine-needle aspiration (FNA) or core biopsy (FNB) as well as for interventional transmural procedures into the surrounding structures, which can be performed in the same session. Endoscopic ultrasound (EUS) is therefore increasingly utilized to investigate various biliary pathologies. Available EUS devices include dedicated echoendoscopes, such as linear array and radial scanning echoendoscopes, and high frequency catheter-based ultrasound probes. Within the wide spectrum of biliary diseases EUS can be utilized as a diagnostic tool for detection of biliary stones Figure 1, in particular microlithiasis [2], D&S of ampullary tumors and evaluation of malignant and indeterminate bile-duct strictures [3,4]. Furthermore, EUS is a distinguished supplement to existing abdominal imaging modalities, such as abdominal ultrasound and cross-sectional imaging, i.e., MRI and CT for the evaluation of gallbladder polyps and staging of malignant masses. EUS has limited ability to visualize intrahepatic portions of the biliary system. However, it can support cross-sectional imaging in staging of tumors by detection of extrahepatic lymphatic adenopathy.

**Figure 2:**
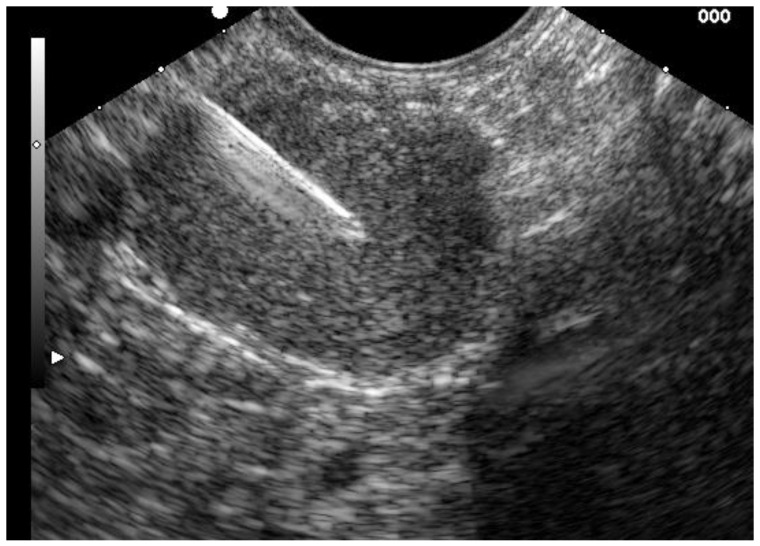
EUS-guided fine needle biopsy (FNB) of a suspicious lymphnode using a dedicated 22 G needle.

Tissue acquisition (EUS-TA) by EUS-guided fine needle aspiration (EUS-FNA) and/or core-biopsy (EUS-FNB) for cytologic and histopathologic examination is nowadays an essential part of the D&S of GI malignancies [3,5]. Figure 2 Advent of novel, dedicated EUS-FNB needles with specialized cutting edge design has made attempts of tissue acquisition suitable for histopathological analysis more effective, which is often needed for individualized oncological treatment based on targeted therapies [3,5]. The common indications for EUS-directed tissue acquisition in biliary diseases include D&S of malignant pancreatobiliary neoplasms or masses and/or adjacent lymphnodes. Nonmalignant processes i.e., inflammatory changes of the bile ducts and or the pancreas, tuberculosis, sarcoidosis, abscesses, and cysts can be also diagnosed through EUS-TA.

ERCP is the therapeutic intervention of choice for biliary drainage in patients with jaundice caused by biliary obstruction. It provides high technical and clinical success rates and a low rate of major adverse events [1,6,7,8]. Failure of ERCP can occur in patients with surgically altered anatomy, inaccessible papilla due to malignancy, or secondary to cannulation failure. Percutaneous drainage has historically been the treatment of choice in patients with failed ERCP [9]. EUS-directed biliary drainage (BD) was first described by Giovannini in 2001 [10]. Since then, EUS-BD has become an alternative to ERCP in cases that require BD [11,12] and in particular as a backup method if standard ERCP-approach fails. Various studies have reported high technical success rates associated with EUS-BD [11,12,13,14,15].

## 2. Materials and Methods

### 2.1. Search for Literature

Aim of this article was to provide an overview of the diagnostic and therapeutic options of endoscopic ultrasound in the context of a personalized approach to bile duct diseases. Search for relevant publications was conducted using the Medline database. Scientific data were mainly extracted from high-quality meta-analyses and prospective and comparative trials, respectively, if available.

### 2.2. Available Echoendoscopes and Technique of EUS Examination of the Biliary System

EUS has many advantages over transabdominal ultrasound, because the abdominal circumference or overlying gas do not impede the quality of the examination. It can be performed using a radial or a linear array echoendoscope. Both systems differ in frequency (7.5–12 MHz vs. 5 or 10 MHZ) and the produced image range (360° cross-sectional image at a right angle vs. images in parallel to the long axis of the endoscope). The radial 360° image is easier to interpretate and therefore recommended for beginners. A handicap of the radial system is the lack of a working channel for biopsies and interventions, respectively, and the reduced scanning depth. The linear array provides a better three-dimensional resolution which helps detecting and delineating smaller lesions and in addition allows a localized deeper tissue penetration. The most important advantage is the working channel allowing EUS-FNA/FNB and other therapeutic interventions. Intraductal ultrasonography (IDUS) uses a thin caliber high frequency ultrasonic probe (12-MHz to 30-MHz), that can be introduced into the bile duct through the papillary orifice [4].

In order to examine the very distal part of the common bile duct (CBD) the echoendoscope has to be placed directly beside the papilla in the second portion of the duodenum while the large remaining part of the CBD can be examined from the upper part of the duodenum.

The right-anterior and -posterior intrahepatic bile duct system as well as the right liver lobe cannot be totally examined by EUS. The left liver lobe and the left intrahepatic arborization of the bile duct can be examined from a position adjacent to the upper gastric lesser curvature. The gallbladder is usually examined from the Antrum or, depending on its position, the duodenum. These aforementioned positions, are used for performing fine needle biopsies with a variety of 19, 22, or 25 G FNA and FNB needles, in different techniques [3,5]. EUS is an inherent part of multimodal pancreaticobiliary imaging. Even in difficult anatomy interventional EUS-procedures can be safely performed under direct real time optical and endosonographical vision through the gastric wall or the duodenum by EUS-needles or electrocautery-enhanced devices, i.e., dedicated cystotomes or delivery systems of lumen-apposing (LA) self-expandable metal stents (SEMS). There are some drawbacks in using EUS techniques. Both diagnostic and therapeutic procedures have a demanding learning curve and patients need sedation due to the large diameter of the EUS endoscope. Alike transabdominal ultrasound there is a significant interobserver variability of this highly operator-dependent procedure.

## 3. Clinical Application and Performance of EUS in Biliary Diseases

Clinical application of endoscopic ultrasound can be subdivided in the field of a diagnostic and therapeutic objective of the procedure. Indications for diagnostic EUS in the biliary tree include evaluation of indeterminate biliary strictures, detection of stones in the biliary system, staging in known cholangiocarcinoma or other malignant neoplasms including lymph nodes, tissue acquisition from strictures and masses in or adjacent to the bile duct system, lymphnodes or liver parenchyma, i.e., in suspected small duct biliary diseases or intrahepatic masses. EUS procedures with a therapeutic objective in the biliary system are predominantly performed to obtain biliary access and drainage, respectively, in most of the cases in malignant diseases. The large variety of possible interventions includes EUS-guided rendezvous procedures, antegrade, transmural stent implantation for a transpapillary drainage, transmural stent implantation for a transmural drainage and facilitating endoscopic access to an endoscopically inaccessible papilla or bilio-enteric anastomosis, respectively, in patients with altered anatomy, i.e., after Roux-en-Y reconstructive surgery with malignant obstruction of the hepato-biliary limb (blind loop syndrome) or after bariatric gastric bypass surgery.

### 3.1. Biliary Stone Disease

Prevalence of biliary stones is approximately 10–15% in Western countries with an overall annual incidence of gallstone development of 0.6% [16]. A prevalence of 8–18% for CBD-stones (CBDS) has been proposed in patients with symptomatic gallbladder stones [17]. There is no data regarding the prevalence of CBDS in asymptomatic patients with gallbladder stones. Major complications like pancreatitis, cholangitis or bile duct obstruction will occur in 1–2% and therefore stone extraction—preferably by performing ERCP—is recommended for symptomatic and asymptomatic patients [1,2]. Risk Patients with gallstones who present with typical symptoms for having CBDS should therefore first of all have transabdominal ultrasound and liver function tests followed by further investigation for confirmation if these initial tests indicate CBDS.

The pretest probability in suspected patients is crucial to determine which patients can benefit best from further assessments. The European Society of Gastrointestinal Endoscopy (ESGE) guideline recommends EUS and MRCP for the diagnosis of CBD-stones in patients with persistent clinical suspicion but with failed proof of CBD-stones on transabdominal ultrasonography [2]. The ability of EUS to detect CBDS has been extensively studied and compared to MRCP as the gold standard radiological method. EUS has been proven to have a higher sensitivity especially in small stones (sensitivity 97% vs. 90% and specificity 87% vs. 92% for EUS and MRCP, respectively) and a significant higher overall diagnostic odds ratio (*p* = 0.008) compared to MRCP in a recent meta-analysis of five comparative trials [18]. Figure 1. Another Meta-Analysis including 18 studies confirmed the high sensitivity and specificity of EUS in the detection of CBDS of 95% (95%CI 91–97%) and 97% (95%CI 94–99%), respectively [19]. For patients with a high pretest probability for CBDS, EUS has the advantage that ERCP can be immediately performed after a positive EUS diagnosis and should therefore be the preferred diagnostic procedure. In a personalized approach to biliary stone disease, EUS can be performed in patients that do present characteristics that may interfere with MRCP, such as claustrophobia, severe obesity, cardiac pacemaker or metal clips. However, MRCP offers potential advantages over EUS because of its non-invasive nature and its capability to display the entire bile duct system. The diagnostic procedure in patients with intermediate risk for CBDS should take individual factors into account. These include patient preference, local expertise, and availability of resources [2,20].

### 3.2. Evaluation of Indeterminate Biliary Strictures

Cholangiocarcinomas can be classified into intrahepatic and extrahepatic tumors. Extrahepatic biliary strictures are challenging for gastroenterologists and surgeons alike. This is due to a broad range of differential diagnoses such as benign strictures of intrinsic or extrinsic origin and malignant intrinsic or extrinsic neoplasms [21]. In 70% to 80% of all primary neoplasias originating from the extrahepatic bile duct, the hilar region with the confluence of both hepatic ducts is involved (perihilar carcinomas) while 20% to 30% of the tumors are located more distally. The cholangiocarcinomas (CCC) located between the papilla and the cystic bile duct junction are defined as distal bile duct tumors [22].

CCC typically present clinically as biliary strictures. However, biliary strictures continue to be a diagnostic challenge because a significant fraction of them remains inconclusive for malignancy despite a thorough multimodal diagnostic approach including radiology, endoscopy and laboratory tests. Premature and exact diagnosis has a major impact on patients’ outcomes in identifying candidates for surgical resection and/or for targeted chemotherapies. The role of EUS in the diagnostic approach to indeterminate biliary strictures and/or suspected extrahepatic CCC, respectively, is still not clear.

Abdominal ultrasound is commonly used as the first line diagnostic modality for suspected liver and bile duct diseases, but the disturbance from overlying gas often prevents reliable diagnostic results in the examination of the distal CBD [22]. CT has a low sensitivity in detecting early tumors [23] in the abdomen and in particular in the biliary tree, while MRI and MRCP are precise and noninvasive modalities for imaging of the hepato-biliary system [24]. Specificity and positive predictive value are not satisfactory as it cannot reliably differentiate between malignant and benign strictures [25]. As a result, usually further extended endoscopic diagnostic procedures are necessary to determine the etiology of a bile duct stricture. The sensitivity of ERCP guided brushing as the standard first line diagnostic procedure in these cases is reported between 27% to 56% [22]. A recent prospective randomized controlled multicenter trial showed significantly higher overall accuracy and sensitivity for detection of malignancy in biliary strictures of forceps biopsies obtained under visual control during digital single-operator Cholangioscopy (d-SOC) of 87.1% and 68.2%, respectively, compared to this standard ERCP approach [26]. However, these favorable outcomes were obtained at expert centers and there is still a diagnostic gap left open that EUS may help to fill, especially as d-SOC is not as widely available as EUS. From the apical duodenum EUS can precisely examine the adjacent extrahepatic portion of the biliary tree and detect bile duct masses which typically present as hypoechogenic lesions. The relationship of the mass towards adjacent structures such as liver parenchyma and arteries as well as the portal vein can be assessed for staging and evaluation of resectability [22]. Endoscopic ultrasound may be utilized to a. staging of a known or suspected mass and b. for tissue acquisition for histopathologic or cytologic diagnosis, respectively [3,5] Figure 2. Accurate presurgical diagnosis is very important in these cases as around 13% to 24% of patients prediagnosed with hilar CCC have benign diseases after surgical resection.

- Local EUS-staging of CCC includes evaluation of the tumor growth pattern and involvement of local lymph nodes. EUS provides high accuracy in terms of local tumor staging of 66–81% and of local lymph node staging of 64–81% and 88–100% in prediction of portal vein infiltration [22]. Intraductal ultrasound seems to provide a high overall accuracy for the local T-staging of cholangiocarcinoma of 92%, whereas its accuracy for staging of lymph node involvement is low with 43% [4].
- The sensitivity and specificity of EUS-based tissue acquisition for the diagnosis of CCC in patients with indeterminate extrahepatic biliary strictures in two recent meta-analyses of 20 and 6 studies, were 66–80% and 97–100%, respectively [21,27]. Another meta-analysis of 10 studies illustrated that EUS was able to improve the detection rate of malignancies in those patients who were investigated for extrahepatic biliary strictures and received a primary non-malignant diagnosis in ERCP by 14% [28]. However, a proximal position of the stricture close to the hilum and indwell of biliary stents may impede the efficacy of EUS-TA in indeterminate extrahepatic biliary strictures. Where possible, EUS-TA should be accomplished directly before ERCP to improve diagnostic yield and staging accuracy in suspected biliary neoplasms. EUS-directed FNA is generally considered safe with low overall rates of adverse events and severe adverse events of 1% and 0.3% [21]. Some authors reported a risk of Needle-track seeding associated with EUS-FNA in hilar CCC whereas this may be of less relevance in distal biliary tumors, as the needle track within the duodenal wall following transmural EUS-FNA is entirely resected during pancreaticoduodenectomy [21].

EUS is an inestimable diagnostic method complementary to ERCP to aid in the identification of a malignant etiology in patients with strictures of the extrahepatic bile ducts. EUS-TA seems to outperform ERCP-based brushing cytology for tissue acquisition in biliary strictures. However, nowadays cholangioscopy-guided forceps biopsy provides similar efficacy. The impact of EUS is possibly higher for patients with strictures located in the distal part of the CBD or those related to compression caused by predominantly extrinsic masses. A recent ESGE guideline suggests EUS-guided sampling for the diagnosis of indeterminate biliary strictures, either as an alternative to or in combination with endoluminal biliary sampling [3].

### 3.3. Endoscopic Ultrasound Guided Biliary Drainage

The established method for endoscopic biliary access and drainage is Endoscopic retrograde cholangiopancreatography (ERCP), for both malignant and benign causes of biliary obstruction. ERCP provides high success rates for biliary cannulation of more than 90% in large series [8,29]. Failure of ERCP can occur in patients with surgically altered anatomy or an inaccessible papilla due to obstruction and/or malignancy, respectively, or secondary to a cannulation failure. Malignant biliary obstruction may involve the ampulla, resulting in a significant drop in success rates of biliary cannulation [30]. Historically, percutaneous transhepatic drainage (PTBD) or surgical bilioenteric anastomosis have been utilized as “rescue” therapies in case of a failed ERCP approach. However, these interventions are associated with significant morbidity and mortality rates [31]. A recent meta-analysis reported equal technical success rates but superior clinical success rates and lower rates of adverse events and quantity of re-interventions in favor of EUS-BD compared to PTBD [9].

#### 3.3.1. Techniques of EUS-Guided Biliary Drainage (EUS-BD)

**Figure 3:**
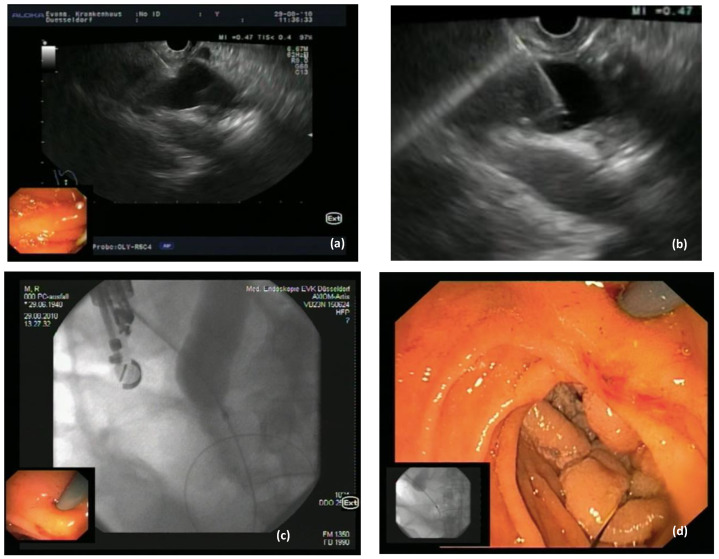
EUS-directed rendezvous procedure in a patient with a malignant duodenal/papillary tumor and unsuccessful cannulation in ERCP. (**a**): dilated common bile duct (CBD) from the duodenum; (**b**) puncture of the CBD using a 19 G EUS needle; (**c**): after contrast injection a 0.035” hydrophilic guidewire was negotiated through the stricture into the duodenum and is now visible in the endoscopic image (**d**) for a rendezvous procedure.

**Figure 4:**
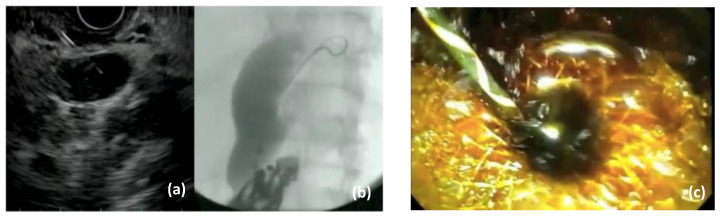
EUS-directed choledochoduodenostomy with (**a**) initial puncture of CBD from the duodenum; (**b**) contrast injection and insertion of a guidewire; (**c**) endoscopic aspect after deployment of a lumen apposing self-expandable metal stent.

**Figure 5:**
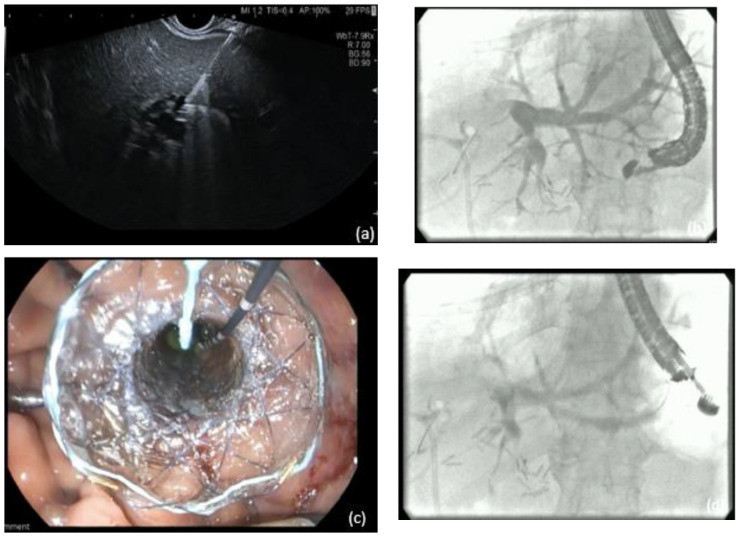
EUS-guided hepaticogastrostomy (EUS-HGA) in malignant hilar obstruction and failure of transpapillary drainage of the left liver lobe. (**a**): EUS guided puncture of a dilated bile duct in segment III; (**b**) insertion of a guidewire to the hilum after aspiration of bile and injection of contrast medium; (**c**) endoscopic aspect of the gastric flange of a dedicated partially covered self-expandable metal stent after deployment into the HGA. (**d**): Fluoroscopic image of the fully expanded stent with subtotal drainage of the contrast medium into the stomach through the stent.

Interventional endoscopic ultrasound in the last decade has become widely available with a variety of indications. EUS is now recommended as a first-line therapy for selected patients in the biliopancreatic system, i.e., drainage of pancreatic collections [32]. There are different approaches for EUS-BD, indication for which depends on the etiology (malignant or benign), location (distal or proximal) of the biliary obstruction and the upper gastrointestinal tract anatomy (surgically altered or not). Prerequisite for endoscopic EUS-BD is the visualization of the dilated extra—and/or intrahepatic bile ducts, respectively, followed by the puncture of the target duct with a needle or a dedicated access device (i.e., LAMS) [33]. Access to the left intrahepatic bile ducts is usually attempted from the oral part of the stomach whereas the optimal position to sight out the CBD is the apical duodenum. Under EUS guidance a wire can be passed into the bile duct and negotiated through the papilla for a rendezvous technique to obtain biliary access [34]. Figure 3. Furthermore, EUS-guided antegrade transpapillary drainage may be performed, i.e., in malignant biliary obstruction. Direct transmural EUS-BD can also be performed for biliary drainage in cases of failed transpapillary access via ERCP [35]. A variety of different transmural approaches for EUS-BD is available, including choledochoduodenostomy (CDS) Figure 4 and hepatogastrostomy (HGS) [14,36] Figure 5. Technical aspects and indications of EUS-BD were recently addressed by consensus guidelines by the Asian EUS group [11]. The choice of the optimal approach as well as the optimal stent (plastic stent, SEMS, LAMS) has to be taken based on the location of the obstruction and extend of ductal dilatation as well as on local expertise. For example, in hilar biliary obstruction, biliary decompression with EUS-CDS is not applicable, as an intrahepatic EUS-BD approach is required. Adverse events associated with EUS-based biliary drainage are bleeding, stent-migration, bile leakage, pneumo (capno-) peritoneum, and peritonitis.

#### 3.3.2. Efficacy and Safety of EUS-BD

A recent meta-analysis of twenty-three studies including 1437 patients found a technical success rate of 92% and a clinical success rate of 87% of EUS-BD using mixed approaches in a variety of indications [13]. Pooled AE rate was reported with 17.9%. The most commonly reported adverse events were biliary leak (4%), infection (3.8%) and stent migration (3.8%). Another meta-analysis of forty-two studies with 1192 patients reported a pooled technical and clinical success rate of EUS-BD of 95% and 92%, respectively [37]. There were no significant differences observed between the two subgroups of a transgastric and a transduodenal approach in terms of technical and clinical success rates as well as of adverse event rates. Another meta-anaylsis found different AE rates of a transduodenal approach and transgastric approach of 14.5% and 20.9% [38]. A recent large meta-analysis reported an overall adverse event rate of 23.3% (bleeding 4.0%, bile leak 4.0%, pneumoperitoneum 3.0%, stent migration 2.7%, cholangitis 2.4%, abdominal pain 1.5%, and peritonitis 1.4%) [37]. A recent retrospective study of EUS-CDS using an electrocautery-enhanced lumen-apposing metal stent (LAMS) for transmural biliary decompression after failed ERCP in malignant biliary strictures showed a technical success rate of 93.5% of which 97.7% showed a clinical success [39]. The adverse event rate of 11.6% including a fatal case is not negligible. However, only 9.3% of the patients need endoscopic re-interventions. Diverging results from different trials may be due to one or more of the following reasons:

(a) Different techniques and routes to access the bile ducts, including hepatogastrostomy (EUS-HG), cholecystostomy, choledochoduodenostomy (EUS-CDD), and other techniques;
(b) Use of different modalities of drainage, such as plastic stents, SEMS, LAMS, nasobiliary drainage tubes, and a combination of these;
(c) The long learning curve with the use of EUS-BD with accumulating experience. A retrospective multicenter trial could show a higher success rate for EUS-BD procedures performed by endoscopists having done more than 500 procedures [40].

#### 3.3.3. EUS-Guided Drainage of the Gallbladder

Endoscopic EUS-guided transmural drainage of the gallbladder (EUS-GBD) is a treatment option for acute cholecystitis in patients with a high risk for complications or contraindications for surgery. In a meta-analysis of twenty-one studies the pooled technical success rate of EUS-GBD was 95.8% whereas clinical response and adverse events were documented in 93.4% and 12.0% of the cases, respectively [41]. Plastic stents had a success rate of 100%, while success rates using SEMSs and LAMS were 98.6% and 91.5%. A clinical success was achieved in 100%, 94.4%, and 90.1% of the cases following implantation of plastic stents, SEMSs, and LAMSs, respectively. It was found that there was 18.2% pooled frequency rate of adverse events using plastic stents. Compared to 12.3% using SEMSs, and 9.9% using LAMSs. Another recent meta-analysis comparing EUS-GBD with two other modalities of interventional drainage of the gallbladder in acute cholecystitis (endoscopic transpapillary and percutaneous drainage) including 10 studies with 1267 patients reported the highest likelihood of technical and clinical success for EUS-GBD and percutaneous drainage while EUS-GBD was demonstrated to be associated with the lowest rate of recurrency of cholecystitis [42].

#### 3.3.4. EUS-Guided Biliary Decompression Ready for Prime Time?

Due to the increasing evidence for the interventional EUS approach, there is a flourishing development to utilize EUS-BD as the initial therapeutic technique for decompression in patients with malignant biliary obstruction, rather than narrowing its use to serve as a backup approach for cases where ERCP fails. A recent ESGE guideline on biliary stenting still recommends ERCP approach as the first line approach for biliary decompression in malignant biliary obstruction [6]. EUS-BD may offer advantages over ERCP in selected patients due to its capability to obtain direct biliary access and decompression (even in cases of difficult papillary access or complete distal obstruction). An instant EUS approach may avoid multiple biliary cannulation attempts in challenging papillary anatomy, therefore promising to decrease rates of post-ERCP pancreatitis (PEP). Available data comparing ERCP and EUS as a primary approach in biliary obstruction is limited and results of studies have to be interpreted with caution because of the low number of cases. A recent meta-analysis of 5 studies including 396 patients reported no significant differences between both techniques as the first line approach in terms of clinical success rates and adverse event rates [12]. While there was a trend in favor of EUS-BD, the PEP rates did not differ significantly.

## 4. Discussion

Endoscopic ultrasound merges investigative and interventional capabilities in a large variety of clinical indications. Available EUS techniques are able to obtain high resolution visualization of the biliary tree and the adjacent structures alike. The ability to acquire tissue and to apply local treatments makes EUS an important invaluable component of the options available for diagnosing, staging, and treating a difficult group of patients with diseases of the biliary system. In the last two decades EUS has evolved from being a solely diagnostic method to that of an interventional modality with a large and still increasing supply of options. After further technical improvements and new developments in scope design, refinements of technique and accessory devices, dedicated stents and probes, EUS has emerged as a reliable backup tool secondary to failure of ERCP in biliarybstruction. Furthermore, EUS directed approach promised advantages over a percutaneous biliary drainage in terms of a significantly lower rate of adverse events and number of re-interventions needed [9]. Thus, EUS may become the first line treatment of biliary decompression in selected patients in the near future, i.e., in patients with malignant distal obstruction and largely dilated extrahepatic bile ducts, if difficult papillary access can be anticipated. Furthermore, in expert hands EUS has been shown to be a safe and effective therapeutic option in selected high risk surgical patients for decompression in acute cholecystitis as well as for facilitating access to the papilla or bilioenteric anastomosis, i.e., in malignant duodenal obstruction, or surgically altered anatomy (i.e., bariatric patients) (EUS-directed trans-enteric ERCP, EDEE [43,44]). Because of a significant learning curve and a considerable rate of adverse events, the future of—in particular interventional—EUS requires significant investment in training programs as well as in development of dedicated accessories, rendering EUS-guided interventions easier and safer. However, given the low quality of currently available evidence, large-scale comparative clinical trials will be needed to define the role that EUS may take in the field of biliopancreatic endoscopy, in particular outside expert centers.

## 5. Conclusions

Endoscopic ultrasound has a huge potential in the clinical management of biliary diseases. Available data shows, that EUS can be considered as the diagnostic method of choice for suspected biliary stone disease, especially for detection of small stones. Furthermore, EUS is an efficient complementary method in the diagnostic workup of known or suspected neoplastic bile duct diseases. EUS-directed tissue acquisition may even be effective after failure of endoluminal cholangioscopically guided brushing or forceps biopsies, respectively. Furthermore, EUS-guided biliary drainage is effective and safe for biliary decompression as a rescue therapy after failed ERCP and as a first line approach in selected patients. However, large scale preferably comparative trials are still needed as well as training programs to translate the favorable results from expert centers into common clinical practice.
